# Point-of-Care Platform for Diagnosis of Venous Thrombosis by Simultaneous Detection of Thrombin Generation and D-Dimer in Human Plasma

**DOI:** 10.1021/acs.analchem.2c03819

**Published:** 2022-12-22

**Authors:** Chunxiao Hu, Valerio F. Annese, Michael P. Barrett, David R. S. Cumming

**Affiliations:** † Division of Electronics and Nanoscale Engineering, James Watt School of Engineering, University of Glasgow, Glasgow G12 8LT, U.K.; ‡ Wellcome Centre for Molecular Parasitology, Institute of Infection, Immunity and Inflammation, University of Glasgow, Glasgow G12 8TA, U.K.

## Abstract

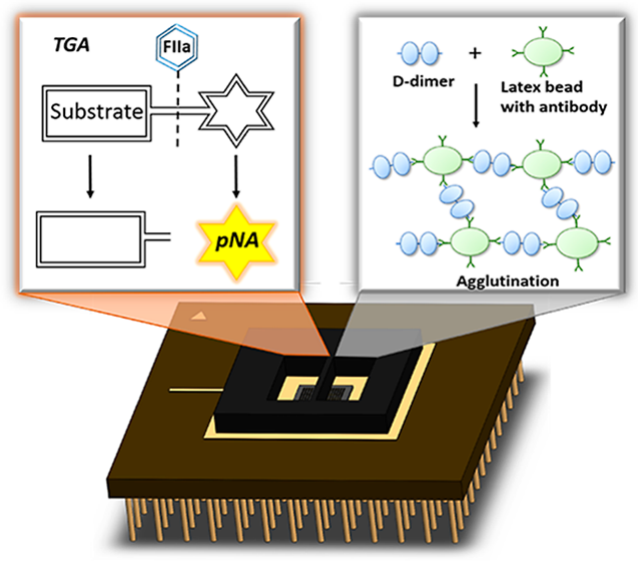

Venous thromboembolism (VTE) refers to a blood clot that starts in a vein. The risk of developing VTE is highest after major surgery or a major injury, or when someone has heart failure, cancer, or infectious disease (e.g., COVID-19). Without prompt treatment to break up clots and prevent more from forming, VTE can restrict or block blood flow and oxygen, which can damage the body tissue or organs. VTE can occur without any obvious signs, and imaging technologies are used. Alternatively rapid measurement of thrombin generation (TG) and D-dimer could be used to make a fast, portable, and easy-to-use diagnostic platform for VTE. Here, we have demonstrated a diagnostic sensing platform with the ability of simultaneous detection of TG and D-dimer in human plasma. Modifications were made to both the assay protocols to eliminate the need for sample dilution and incubation steps. Using a substantially reduced sample volume, the measurement results show comparable performance to the gold standard method. Our platform is able to deliver accurate and cost-effective results for both TG and D-dimer assays when using undiluted plasma in under 15 min. The assays presented are therefore a good candidate technology for use in a point-of-care platform to diagnose VTE.

Venous thromboembolism (VTE), also known as blood clots, is the blockage of a vein caused by a thrombus.^1^ VTE is among the most common causes of vascular death after heart attack and stroke.^2^ According to the American Heart Association, it affects between 300,000 and 600,000 people per year in America and bears a significant cost burden.^3^ VTE is a disorder that includes deep vein thrombosis (DVT) and pulmonary embolism (PE). DVT is a clot in a deep vein, usually in the leg but sometimes affects the arm or other veins. PE occurs when a DVT clot breaks free from a vein wall, travels to the lungs, and then blocks some or all of the blood supply.^1^ The most common triggers for VTE are surgery, cancer, immobilization, and hospitalization. Individual patient factors, current disease state, recent or planned surgical procedures, and underlying hematologic disorders all add up to give a patient risk of VTE.^3^ VTE in patients having their first experience occurs at a rate of 100 persons per 100,000 each year in the United States, and it recurs frequently in the first few months after the initial event, with a recurrence rate of 7% at 6 months. Death occurs in 6% of DVT cases and 12% of PE cases within 1 month of diagnosis.^4^ The estimated cost of VTE in the US is $10 billion in 2016, with each episode estimated to cost between $9407 and $28,353 for VTE and $11,486 to $19,901 for PE. Recurrent events incurred reported costs of up to $82,110 in combined inpatient and outpatient costs.^5^ Therefore, a device can be used for early diagnosis, and regular monitoring of VTE is needed.

In some cases, VTE displays several signs and conditions. For example, leg pain, leg swelling, and reddish discoloration are symptoms of DVT, and unexplained shortness of breath, chest pain, and fast heart rate are exhibited for PE.^1^ However, those symptoms can also be indicators for other diseases such as muscle injury, cellulitis, heart attack, and pneumonia.^6^ In some other cases, VTE patients may not produce any symptoms. Therefore, special tests that can look for clots in the veins or in the lungs are needed to diagnose. In patients with suspected VTE, the goal of diagnosis is to rapidly and accurately distinguish those with the condition from those without it. This is essential because patients with VTE require rapid initiation of anticoagulant therapy, whereas those without the condition do not. The wrong diagnosis is problematic because failure to prescribe anticoagulant therapy to patients with VTE can enable thrombus extension and fatal PE and because inappropriate administration of anticoagulants to those without VTE can lead to fatal bleeding. Rapid and accurate diagnosis is therefore essential.^7^

Conventional diagnostic methods for DVT include compression ultrasound and CT pulmonary angiography.^8^ Other less frequently used methods include ventilation-perfusion lung scanning that is used for the diagnosis of PE.^7^ The challenge with the reliance on imaging for ruling out VTE is the high false positive rate of the current early diagnosis methods, and most patients with suspected VTE do not actually have it. The diagnosis is confirmed in only about 20 and 5%, respectively, of those with suspected DVT and PE. Relying on imaging in patients, however, is problematic. It increases the cost to health-care systems, adds the unnecessary exposure to radiation for the majority of patients with suspected PE, and has the potential for over-diagnosis of events of uncertain clinical significance, such as calf DVT and subsegmental PE.^7^ This problem was overcome by combining the pre-test probability with a sensitive assay for measurement of the level of D-dimer in plasma.^9^ D-dimer is a plasmin-derived breakdown product of cross-linked fibrin. D-dimer levels are elevated in most patients with VTE,^7,10,11^ and it remains the only biomarker in routine clinical use.^12^

In addition to measuring D-dimer, research has recently shown that parameters associated with the thrombin generation (TG) potential are strongly associated with the risk of VTE.^13−16^ A TG assay (TGA) is a global coagulation assay that can be used to assess coagulation and thrombotic risk.^17^ It is based on the potential of a plasma to generate thrombin over time, following activation of coagulation.^18^ As a potential indicator for VTE, the combined monitoring of changes in TG and D-dimer concentrations has been used in some applications such as for women injecting enoxaparin during pregnancy and the puerperium.^19^ More recently, it has been discovered that these measurements can also predict major adverse events in COVID-19.^20^

For the applications mentioned above, conventional methods for measuring the TG and D-dimer levels were used. Central laboratory quantitative D-dimer assays were initially based on an enzyme-linked immunosorbent assay (ELISA) technology but have more recently been adapted to use coagulation and clinical chemistry analyzers with an endpoint based on immunofluorescence,^21^ latex-enhanced immunoturbidimetry,^22^ or chemiluminescence.^23^ Although these assays are highly sensitive and are economical to perform when analyzing large numbers of specimens, the combination of specimen transportation time and analytical time often results in a prolonged response time of more than 40 min.^24^

Point-of-care D-dimer latex assay was developed to simplify the diagnostic process and shorten the time.^25^ It was a rapid agglutination assay utilizing latex beads coupled with a highly specific D-dimer monoclonal antibody. It reduced the assay time to less than 10 min but required manual operation and was only semi-quantitative. Our new assay was implemented to work using plasma since it is widely used in the field of application. Using plasma also helps improve the sensitivity when using optical sensors when compared to working with, for example, whole blood.^26^

For TGA, there are three commercially available TG methods at present, based on calibrated automated thrombography (CAT).^27^ Two of these methods are based on fluorogenic assays with a scan time of 50–120 min, and third one is chromogenic assay with a scan time of 20 min. Although researchers have explored the possibility of developing a point-of-care TGA,^28,29^ currently no device has been developed. There is an increasing need for a point-of-care device that can simultaneously detect D-dimer and TG.

In this paper, we demonstrated a digital, low cost handheld sensing platform for diagnosis of VTE in a stand-alone point-of-care technology format. It is designed to allow the rapid and simultaneous measurement of D-dimer and TG from a single drop of human plasma. D-dimer assay we present was modified from D-dimer latex agglutination assay. Instead of estimating the concentration level by diluting the plasma sample, our platform provides a quantitative reading. The TGA measurement we present is underpinned by the available chromogenic assay, but modifications were implemented to remove complicated operating steps: no dilution of the sample is required, no incubation steps are involved, and minimal training is required for the operator. The platform combines a silicon complementary metal oxide semiconductor (CMOS) photodiode (PD) sensor chip, a measurement cartridge, a reader, and a computing platform with graphical user interface (e.g., PC or tablet). The latex agglutination from D-dimer assay and the color change generated from TGA were simultaneously measured and recorded. Our results show comparable performance to the gold standard method and hence the new sensing platform as a candidate for diagnosis of venous thrombosis in point-of-care settings.

## Materials and Methods

In this paper, we demonstrate the implementation of TG and D-dimer assays on a chip-based platform so that the final device is compact and capable of completing both measurements in only 15 min.

### Thrombin Generation Assay

TGA can provide an overall assessment of hemostasis by detecting the thrombin level generated during the blood coagulation process.^30,31^ There are two common analysis methods for TGA: fluorogenic and chromogenic.^27^ We chose the chromogenic method since it has a lower scan time (20 min) when compared to fluorogenic (50–120 min). The chromogenic method is also well-matched to the chip-based technology we have adopted.

The main components for the chromogenic TGA include tissue factor (TF), chromogenic substrate, fibrin inhibitor, calcium ions (CaCl_2_), Tris–HCl buffer, and plasma sample. In the conventional chromogenic TGA experiment, the plasma sample is added first and platelet poor plasma is used; thus, the method is not practical for a point-of-care application.^27,32^ To overcome this problem, we have modified the protocol to make it possible to only add the patient plasma sample as the final step without further centrifugation. To achieve that, the assay volume was reduced from 260 to under 10 μL, and a relatively high concentration of fibrin inhibitor was used.

Prior to the measurement, a mixed solution of the chromogenic substrate (5 mM, 100 μL), TF (6 nM, 80 μL), fibrin inhibitor (36 mg/mL, 25 μL), CaCl_2_ solution (250 mM, 25 μL), and Tris–HCl buffer (50 mM, pH 7.4, 100 μL) was prepared in an Eppendorf tube. 6 μL of the mixed solution was preloaded to the reaction zone on the sensor chip surface. For the actual measurement, the first step is to start recording data using the software to obtain a baseline signal. The next step is to add 4 μL of the human plasma sample to initiate the reaction. A total of 20 min signal is recorded, and all the measurements were undertaken in the dark.

### D-Dimer Assay

As previously discussed, instead of ELISA, a rapid latex agglutination assay for the D-dimer measurement was modified and used in this research. The main components for the assay include D-dimer latex (latex beads that are coated with anti-D-dimer monoclonal antibody), saline solution, and a plasma sample. The conventional assay is a semi-quantitative method, which involves serial dilution of the plasma samples by 1:2, 1:4, 1:8, 1:16, 1:32, and 1:64 with saline solution using small test tubes, manual mixing of the latex suspension and the sample solution, and naked eye observation of the agglutination on a test card with a timer (see Supporting Information Figure S1).

In our measurement, none of those operations are required, and a quantitative reading can be achieved within 20 min. Prior to the measurement, a vial of latex beads, coated with anti-D-dimer monoclonal antibody and suspended in Hepes buffer, pH 8.2, with 0.2 g/L sodium azide as preservative, was agitated by repeatedly inverting the vial to disperse sedimented latex particles. Prior to the measurement, 4 μL of the latex bead suspension was preloaded in the reaction zone on the sensor surface. Similar to the TGA measurement, the first step is to start recording on software to obtain a baseline signal and followed by adding 6 μL of the human plasma sample to initiate the agglutination process. All the measurements were undertaken in the dark.

### Chemical Preparation

The BIOPHEN plasma calibrator (222101) and BIOPHEN normal control plasma (223201) were purchased from Quadratech Diagnostics. TEClot Factor VIII Deficient Plasma, Teco (P5301-010) was sourced from Alpha Laboratory. TG chromogenic substrate (T3068), human D-dimer ELISA kit (RAB0648), alpha 2 macroglobulin from human plasma (SRP6314), fibrin polymerization inhibitor Gly–Pro–Arg–Pro (G1895), thrombin from human plasma (T6884), calcium chloride (C1016), Trizma hydrochloride (T3253), bovine serum albumin (A7030), and sodium azide (S2002) were bought from Sigma-Aldrich (U.K.). Recombinant human coagulation factor III/TF (2339-PA) was purchased from R&D Systems.

The TECO D-dimer agglutination kit (D2050-000) was purchased from Alpha Laboratories Ltd. It includes one vial containing 1.7 mL of suspension of latex beads which are coated with anti-D-dimer monoclonal antibody and suspended in Hepes buffer, pH 8.2, with 0.2 g/L sodium azide as preservative; two vials of 8 mL of saline solution, pH 7.3, with 0.2 g/L sodium azide; one vial containing 0.2 mL of lyophilized human plasma enriched with fibrin D-dimer; one vial containing 0.2 mL of lyophilized human plasma; 16 test cards; and 50 mixing sticks.

Polydimethylsiloxane or PDMS (SYLGARD 184 silicone elastomer kit) was purchased from Dow Corning. Black epoxy (302-3M Black) was purchased from Epoxy Technology, Inc. Black epoxy was prepared by mixing the two parts of the product (part A & B) with a ratio of 100:45 by weight. It was left at room temperature (20 °C) for 24 h to cure.

### CMOS Sensing Platform

The assay materials were preloaded in the reaction zones on to the chip prior to the measurement, so that all measurements were collected electronically. The entire sensing platform is a handheld device with cable connection to a computing platform.^33−35^ It is based on a 16 × 16 array of PDs which was designed and fabricated using a commercially available CMOS 350 nm high voltage 4-metal process. The array can be used to exploit the statistical method of averaging signals from independent Gaussian noise sources, either over time or spatially, to improve the overall system sensitivity. A LED mounted in a 3D-printed housing was used as a light source so that the LED was immediately over the sensing area of the chip. The housing also doubled up as a light-proof unit to eliminate unwanted stray light from the measurements. A wavelength of 405 nm is recommended for the chromogenic substrate, but silicon PDs are relatively insensitive at this wavelength. After testing at 405, 430, and 450 nm, 430 nm was found to be the best choice for TGA. The wavelengths used in the literature for latex agglutination varies from 340 to 490 nm.^36−38^ Since not much difference was found among those wavelengths, 430 nm was also selected for D-dimer assay.

**Figure 1:**
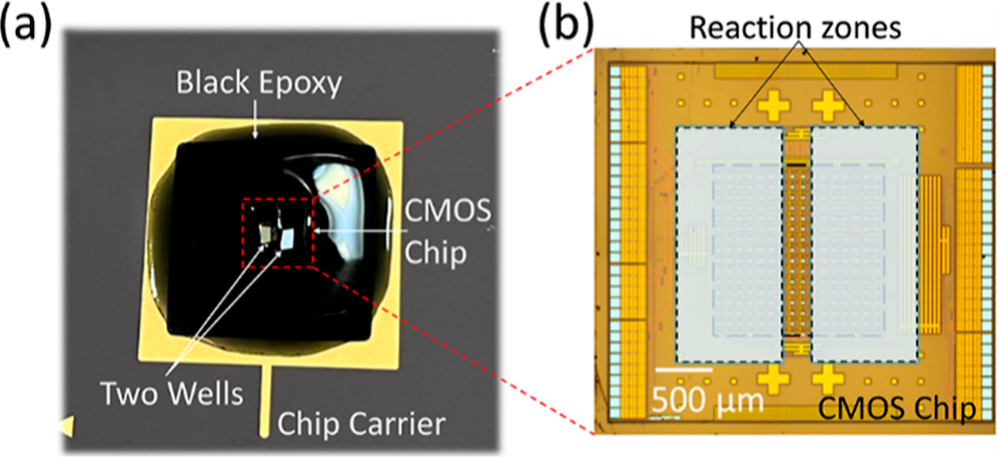
Cartridge of the sensing platform. (a) Photograph of a cartridge including a chip carrier, a CMOS chip, and two black epoxy formed reaction wells. (b) Enlarged view of the CMOS chip. The two reaction zones are located on the top of the sensing area.

The size of the entire CMOS chip is 3.4 × 3.6 mm with a sensitive area of 1.6 × 1.6 mm. The sensitive area of the sensor array was divided into two independent regions accommodating D-dimer latex agglutination assay and TGA chromogenic assay, as shown in Figure 1. To fabricate the two independent reaction zones on the sensing area, the CMOS chip was wire-bonded on to a ceramic 120 pin grid array chip carrier. Two sacrificial PDMS micro-blocks with a size of 0.8 mm × 2 mm and height of 1.5 mm were temporarily attached on to the sensitive area of the CMOS-chip with a gap of 0.2 mm. Black epoxy was prepared and carefully micro-pipetted over the area of the CMOS chip and the contact pads. After the epoxy resin was cured at room temperature (20 °C) for 24 h, the PDMS micro-blocks were removed to leave two microwells exposed as reaction zones^39^

### Data Acquisition and Analysis

The reader provides functionality for on-chip sensor multiplexing and addressing, data digitization, and transmission to a personal computing device via a USB link. The reader is programmed before use with custom firmware. Data are digitized using the embedded 12-bit analogue to digital converter with an average rate of 36 frames per second (add reference to where we have described this in more detail before).

For TGA, the relation between velocity and thrombin concentration varies during the experiment because the substrate is consumed, and color change is not linear with the concentration of the product.^40^ Therefore, a TG curve is usually extracted from the raw data to provide more useful information. A TG curve is characterized by an initiation phase (lag-time) followed by the formation of large amounts of thrombin (propagation), culminating in a peak thrombin concentration, and finally inhibition of TG by natural anticoagulants. The endogen thrombin potential (ETP), which is the area under the curve, represents the amount of thrombin formed during the reaction.^30^ To obtain a TG curve from the raw data, we wrote a Matlab program to extract the reaction rate and provide a new evaluation once a second. With chromogenic substrates, one measures the combined amidolytic activity of free thrombin and the α_2_M-thrombin complex. α _2_M-thrombin is formed during TG from free thrombin and plasmatic α_2_M in a reaction that is of pseudo-first order, and the velocity with which α_2_M-thrombin is formed is proportional to the concentration of thrombin.^40^ Therefore, the impact of α_2_M-thrombin can be deducted mathematically, which was also applied in this research (Supporting Information Figure S2). The lag time, peak potential, and ETP were extracted from the TG curve.

In D-dimer assay, the velocity and amount of latex bead agglutination are proportional to the concentration of D-dimer in the plasma sample up to 25 min from commencement. Therefore, both the end-point value and the initial reaction rate can be used for characterizing the calibration curve.

The point when the plasma sample was added was counted as time *t* = 0, and the signal change due to the reaction was recorded in real time. The change of the signal with respect to time in a fixed interval gave the reaction rate, which was calculated by dividing the magnitude of the signal change by the corresponding time. The limit of detection (LOD) was quantified using the “International Union of Pure and Applied Chemistry” (IUPAC) definition. The average (μ_c_) and standard deviation (σ) of the initial reaction rate for negative controls (common to all the assays) were calculated, and consequently, the LOD (μ_c_ + 3.3·σ_c_) was obtained.

## Results

### Results from Individual Assay

Individual assays were carried out separately for TGA and D-dimer on the sensing platform to validate their performance.

**Figure 2:**
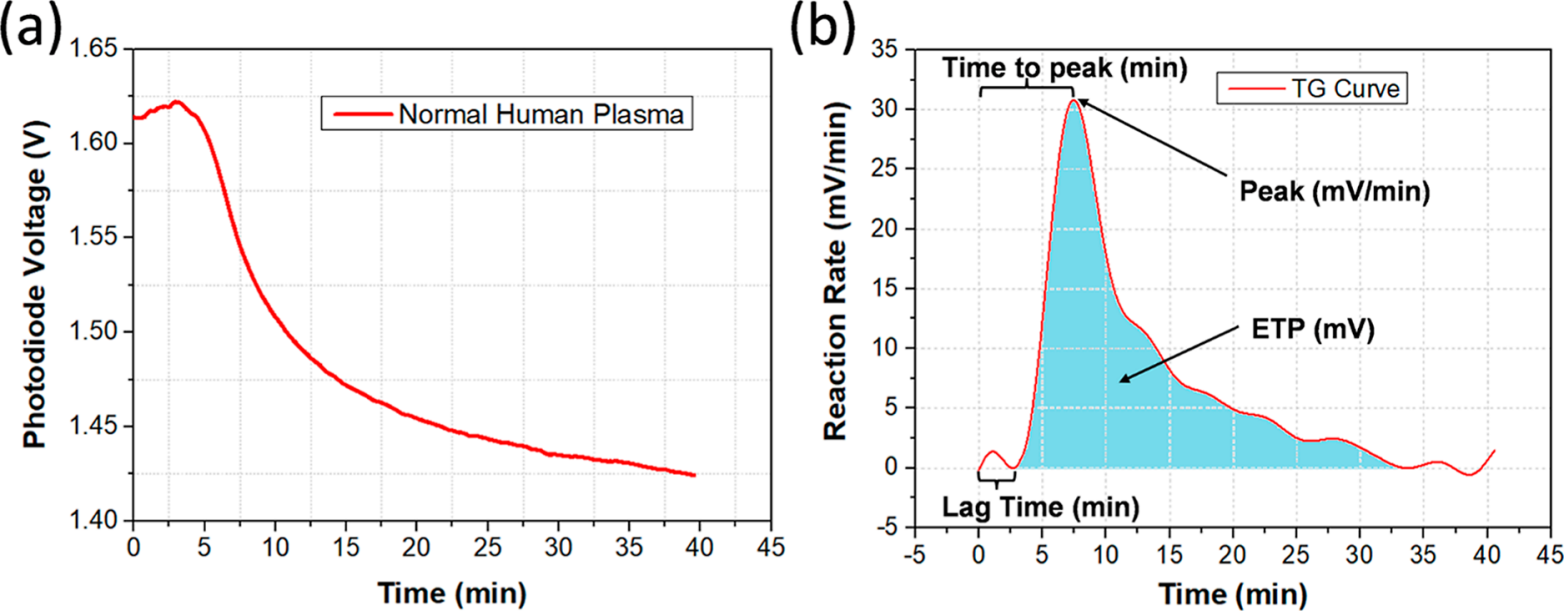
Results from TGA. (a) Reaction curve from an undiluted human plasma sample. (b) TG curve derived from the reaction curve in (a). All the parameters such as lag time, time to peak, peak height, and ETP can be clearly observed and recorded.

The first measurement was TGA using the modified chromogenic method. As mentioned in the previous section, the plasma sample in the conventional chromogenic assay is required to be present in the first step to initiate the assay. This is not suitable for a point-of-care diagnostic device that requires the sample to be introduced as the final, and only, user step. As discussed, modifications to the protocol were therefore implemented to make it possible to add the plasma sample as the final step on our sensing platform. As shown in Figure 2a, undiluted human plasma was used as the test sample for the TGA measurement on our platform. The chromogenic substrate changed its color from clear to a brownish hue during the reaction; thus, a decrease in the signal was observed as expected since the light transmission through the reaction solution reduced. The degree of signal change is related to the thrombin generated during the assay. Time 0 was the point that the plasma sample was added to the reaction zone on the sensor surface. A TG curve was successfully extracted from the reaction curve (Figure 2b), which is comparable to that in the literature. It contains all the parameters such as lag time, time to peak, peak height, and ETP that is required for the characterization of the plasma sample. As described in the Data Analysis Section, the impact of α_2_M-thrombin was subtracted mathematically (Supporting Information Figure S2). A total of 40 min of data was recorded to better cover the entire TG process.

**Figure 3:**
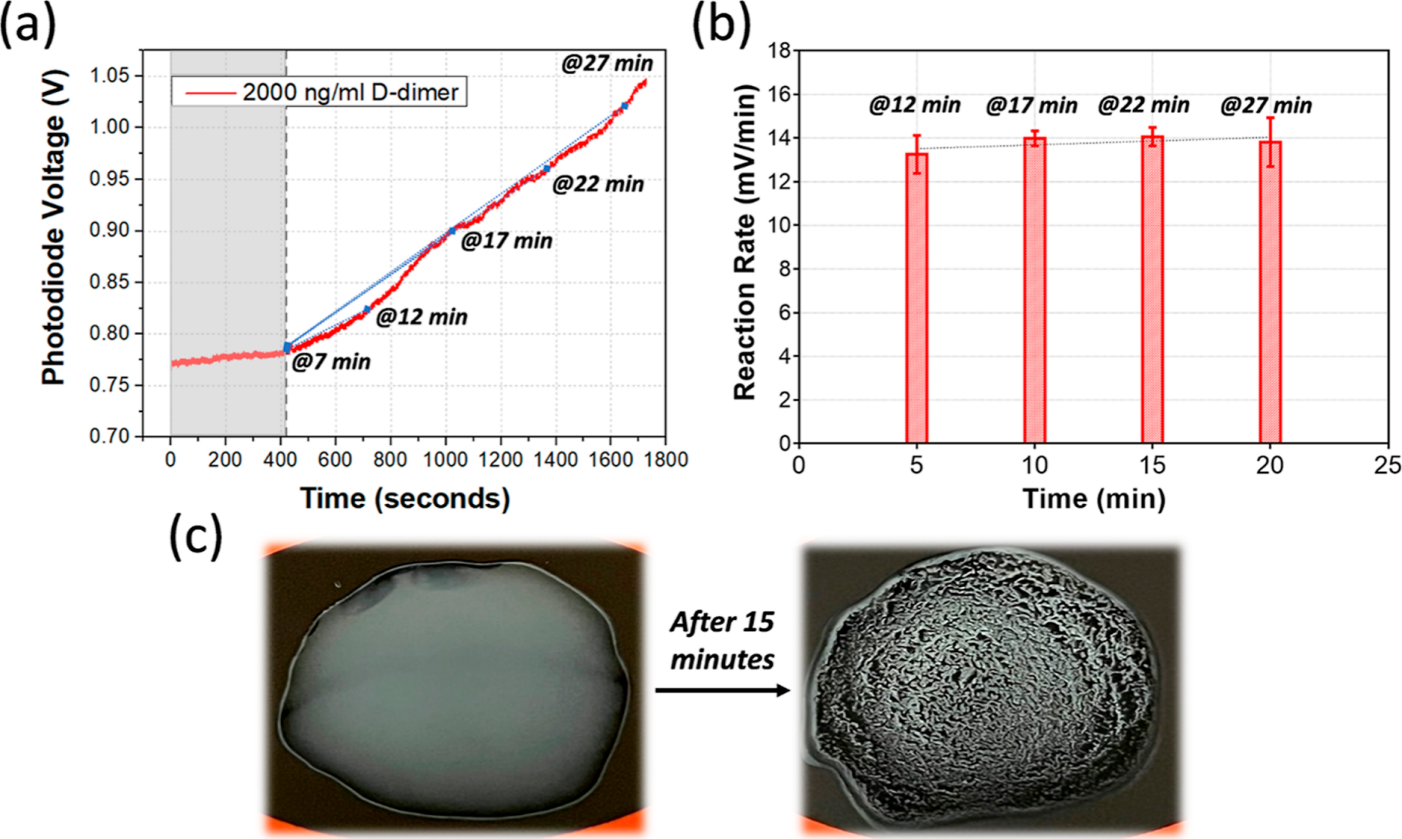
Results from D-dimer assay. (a) Reaction curve from human plasma sample spiked with 2000 ng/mL D-dimer. The first 420 s (gray area) was considered as the diffusion period. (b) Reaction rates for the first 5, 10, 15, and 20 min (@7 min were considered as the start point of the reaction). (c) Image of the solution before and 15 min after the reaction.

D-dimer assay was also conducted individually on the platform using the latex agglutination method. The TECO D-dimer agglutination kit was used here to validate the performance of our sensing platform. Conventional D-dimer agglutination assay was first undertaken following the instruction from the manufacturer (Supporting Information Figure S1). The latex beads are coated with anti-D-dimer monoclonal antibody, which attach to the D-dimer in the sample. Therefore, latex bead agglutination was observed for the sample with high concentration of the D-dimer. As shown in Figure 3a, a plasma sample with 2000 ng/mL D-dimer was used as the test object. The suspension of the latex beads naturally blocked most of the light transmitting through the solution on to the sensor surface. After adding the plasma sample, it took around 7 min for the reaction to occur. This is because in the conventional method, the solution is continuously agitated for 3 min, which was not the case in the present work. We have simplified the operation to just add one drop of the sample. At 7 min, the reaction was considered to begin since no obvious signal change was observed before that, and a signal increase was observed afterward, owing to latex bead agglutination (Figure 3a). As the beads agglutinated, a clear area appeared in the solution, which allowed more light to pass through (Figure 3c). When the reaction started, a linear response was observed for the following 20 min (Figure 3a). Therefore, instead of measuring the end-point value (Supporting Information Figure S3), the reaction rate was calculated within the initial linear phase in order to collect sample-specific data in less time. The reaction rates were calculated every 5 min after the initial reaction to find out the best time point to be used in the assay. Since an elapsed time of 7 min was used as the reaction start time, time 0, the reaction rates at 12, 17, 22, and 27 min were obtained (Figure 3b). A similar reaction rate was found for each of the four time points, but the measurement at 17 min was found to show the best performance with the smallest standard deviation.

### Results from the Simultaneous Measurement

Both TGA and D-dimer assays were successfully performed on the sensing platform individually, thus providing the foundation for making a simultaneous measurement that would be valuable to a future diagnostic device.

**Figure 4:**
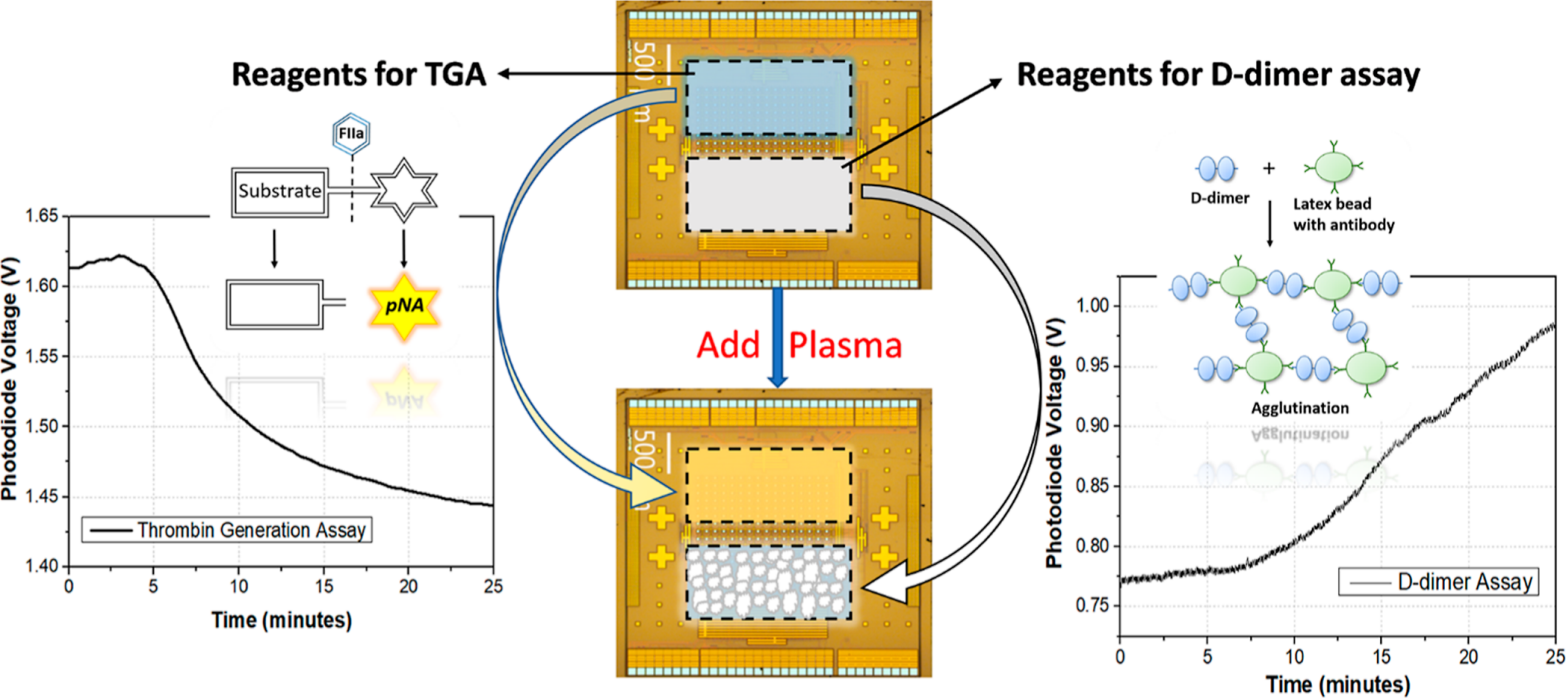
Working principle of simultaneous detection of TG and D-dimer. The two reaction zones on the CMOS chip were filled with reagents for the two assays, respectively, prior to the measurement. Plasma sample was added to the two wells to initiate the two assays. The signals from both assays can be observed and recorded simultaneously.

As shown in Figure 4, the reagents for TGA and D-dimer assay were pipetted in the two reaction wells on the sensor surface prior to the actual measurement. The factor VIII-deficient plasma was spiked with the factor VIII plasma calibrator (includes 100 IU/dL factor VIII) to mimic hemophilia patient plasma samples for the measurement of TGA. Ideally, there should no factor VIII inside the factor VIII-deficient plasma, but there is always a very low concentration of factor VIII. The depleted factor VIII used in this work contained 0.5 IU/dL factor VIII. Since there is also no D-dimer in the factor VIII-deficient plasma, the same plasma sample was also spiked with the D-dimer (include 4000 ng/mL D-dimer) for the measurement of D-dimer assay. A plasma sample with 50 IU/dL factor VIII and 500 ng/mL D-dimer was made to give the highest concentration. It was then diluted with the factor VIII-deficient plasma to produce another four plasma samples, with concentrations of factor VIII of 0.5, 6.25, 12.5, and 25 IU/dL, and D-dimer of 0, 62.5, 125, and 250 ng/mL, respectively. The five plasma samples were measured in order of increasing concentration with the sensing platform for simultaneous measurements of TGA and D-dimer assay. Once the plasma sample was added to the two reaction wells, two reaction curves were observed and recorded at the same time (Figure 4). As described in the previous section, the chromogenic TGA produces a color change from clear to brownish; thus, a signal drop was observed, and the latex agglutination D-dimer assay generates clear regions, from which a signal increase was seen. The parameters of the TG curves from plasma samples with different concentrations of factor VIII were calculated and are listed in Table 1. No obvious cross-talk issue was found during the measurements (Supporting Information Figure S4).

**Table 1:** Parameters of the TG Curves from Plasma Samples with Different Concentrations of Factor VIII

	0.5 IU/dL FVIII	6.25 IU/dL FVIII	12.5 IU/dL FVIII	25 IU/dL FVIII	50 IU/dL FVIII
lag time (min)	9.8 ± 0.17	5.0 ± 0.11	4.2 ± 0.1	3.4 ± 0.14	2.7 ± 0.11
peak time (min)	18.9 ± 0.91	13.9 ± 0.71	10.8 ± 0.36	8.5 ± 0.22	6.5 ± 0.12
peak height (mv/min)	2.8 ± 0.33	8.5 ± 0.45	15.9 ± 1.93	23.1 ± 0.92	30 ± 0.75
ETP (mV)	28.6 ± 4.3	81.4 ± 6.5	125 ± 5.9	159.8 ± 8.0	187.0 ± 4.5

**Figure 5:**
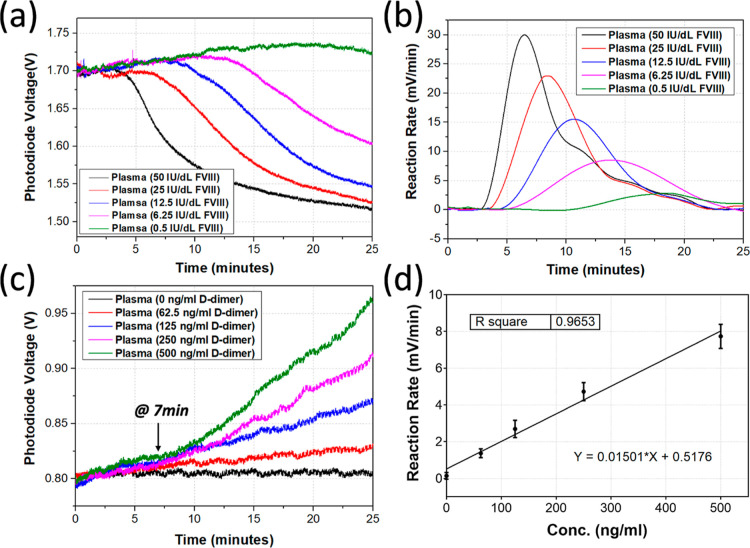
Results from the simultaneous measurements. (a) Reaction curves from TGA with factor VIII-deficient plasma samples spiked with factor VIII control plasma to achieve 0.5, 6.25, 12.5, 25, and 50 IU/dL factor VIII plasma samples. (b) TG curves derived from TGA reaction curves from (a). (c) Reaction curves from D-dimer assay with factor VIII-deficient plasma samples spiked with D-dimer-rich plasma to achieve 0 to 500 ng/mL D-dimer plasma samples. (d) Reaction rates of D-dimer assay for the concentrations between 0 and 500 ng/mL that provides a linear response.

The results from the simultaneous measurements for TGA and D-dimer assay with five different concentrations are plotted in Figure 5. Each measurement was recorded for 25 min, which was enough to provide the information required for analysis. TG curves (Figure 5b) were produced from TGA reaction curves (Figure 5a), and all the parameters including lag time, peak time, peak height, and ETP were measured and are listed in Table 1. For D-dimer assay, as described previously, the reaction rate at 22 min (Figure 5d) was used to characterize the result (Figure 5c). A linear curve with *R*^2^ of 0.9653 was obtained for the D-dimer concentration below 500 ng/mL, which is good enough to cover the physiological range, as the D-dimer concentration for health person is usually below 200 ng/mL. The signal is slightly noisier from D-dimer assay, which is probably due to the agglutination of the latex beads and their motion. However, it does not strongly affect the sensitivity.

## Conclusions

We have successfully demonstrated a potential point-of-care platform for diagnosis of venous thrombosis by simultaneous detection of TG and D-dimer in human plasma. Conventional TGA (chromogenic) and D-dimer (latex-bead agglutination) assays were required to be modified to create a format suitable for point-of-care use. With the proper modifications to the assay protocols, the new assay methods provided many advantages over the conventional methods: no dilution or defibrination of the sample is required, no incubation steps are involved, the assay time is lower, assay volume is dramatically reduced, the sample is added as the final step, no bulky equipment is used, and minimal training is required for the user. Our results show comparable performance to the gold standard method (Supporting Information Table S1), which makes our sensing platform a potential candidate for diagnosis of venous thrombosis. The device also demonstrated good reproductivity and stability (Supporting Information Figure S5).

The dual assay technique is fast at <15 min and has the potential to support the early diagnosis of VTE with reduced delay, improving patient outcomes. As discussed, the risk of developing VTE is highest after major surgery, a major trauma, after heart failure or a heart attack, and in patients with cancer or an infectious disease (e.g., COVID-19). A prompt diagnosis of VTE under these circumstances using the proposed point-of-care device has the potential to be lifesaving and enables healthcare workers to make high-quality decisions and use resources more efficiently.

To convert the research prototype into a diagnostic device will require future work to introduce a sample flow technology such as microfluidics on to the chip-based format to carry the patient sample on to the sensor. Additional potential biomarkers for VTE (such as factor VIII, P-selectin, and C-reactive protein) could in future be included and measured simultaneously on the platform to increase the sensitivity and selectivity of the test. Clinical trials using patients with venous thrombosis disorders and control groups are required to further validate our sensing platform.

## Supporting Information Available

Conventional D-dimer agglutination assay on the testing card (a semi-quantitative method); impact of α_2_M-thrombin subtracted mathematically for the TG curve; results from D-dimer assay with concentrations from 0 to 2000 ng/mL; cross-talk tests were undertaken to check the performance of the two reaction zones; reproductivity and stability of the device; and comparison of the diagnostic methods for VTE (PDF)
