# Supplementary material for: Point-of-Care Platform for Diagnosis of Venous Thrombosis by Simultaneous Detection of Thrombin Generation and D-Dimer in Human Plasma
Source: Anal Chem. 2022 Dec 22;95(2):1115–22. doi: 10.1021/acs.analchem.2c03819 (PMC9850404; doi:10.1021/acs.analchem.2c03819)
Supplement: Supplementary file 1 — ac2c03819_si_001.pdf [file ac2c03819_si_001.pdf]

Point of care platform for diagnosis of venous thrombosis by simultaneous detection of thrombin generation and D-dimer in human plasma

Chunxiao Hu†*, Valerio F. Annese†, Michael P. Barrett‡ and David R. S. Cumming†

† Division of Electronics and Nanoscale Engineering, James Watt School of Engineering, University of Glasgow, United Kingdom. G12 8LT

‡ Wellcome Centre for Molecular Parasitology, Institute of Infection, Immunity and Inflammation, University of Glasgow, United Kingdom. G12 8TA

Corresponding author: Chunxiao Hu, Chunxiao.hu@glasgow.ac.uk

Table of content

Supporting Figures……………………………………………………………..S-1

1. The conventional D-Dimer Agglutination assay on testing card is a semi-quantitative method…………………………………………………..…………...……......S-2
2. The impact of α_2M-thrombin is subtracted mathematically for the TG curve..…..............…S-3
3. Results from the D-dimer assay with concentrations from 0 to 2000 ng/ml……..................S-4
4. Cross-talk tests were undertaken to check the performance of the two reaction zones……………………………………………………...……….…………S-4
5. Reproductivity and stability of the device……………………………………………S-5

Supporting Tables…………………………………………………….………..S-5

1. Comparison of the diagnostic methods for VTE…………………………….…...…....S-5

(a)

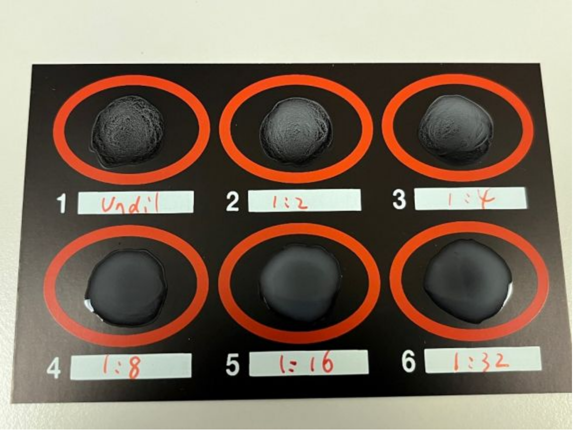

(b) D-dimer Level, ELISA scale, ng/mL

ng/mL	Undil.	1:2	1:4	1:8	1:16	1:32	1:64
< 250	-	-	-	-	-	-	-
250 -500	+	-	-	-	-	-	-
500 -1000	+	+	-	-	-	-	-
1000 -2000	+	+	+	-	-	-	-
2000 -4000	+	+	+	+	-	-	-
4000 -8000	+	+	+	+	+	-	-
8000-16000	+	+	+	+	+	+	-
> 16000	+	+	+	+	+	+	+

Plasma or serum containing more than 250ng/mL D-dimer gives an agglutination pattern represented by (+).

Figure S1: The conventional D-Dimer Agglutination assay on testing card is a semi-quantitative method. (a) Results on a test card. 100 µl of sample (human plasma contains 4000 ng.ml D-dimer) was serially diluted by 1:2, 1:4, 1:8, 1:16, and 1:32 with 100 µl saline solution using small test tubes. Positions were marked for the different dilutions and the samples (20 µl) were added accordingly. 20 µl of a latex bead suspension was added beside the samples and they were mixed together. The card was then continuously agitated for 180 to 200 seconds and observed. (b) The ranges of semi-quantitatively read D-dimer concentration (positive (+) or negative (-) agglutination is compared to results obtained using the controls.). If agglutination is observed, a pathological condition probably exists. Plasma containing more than 250ng/mL D-dimer gives an agglutination pattern represented by (+).

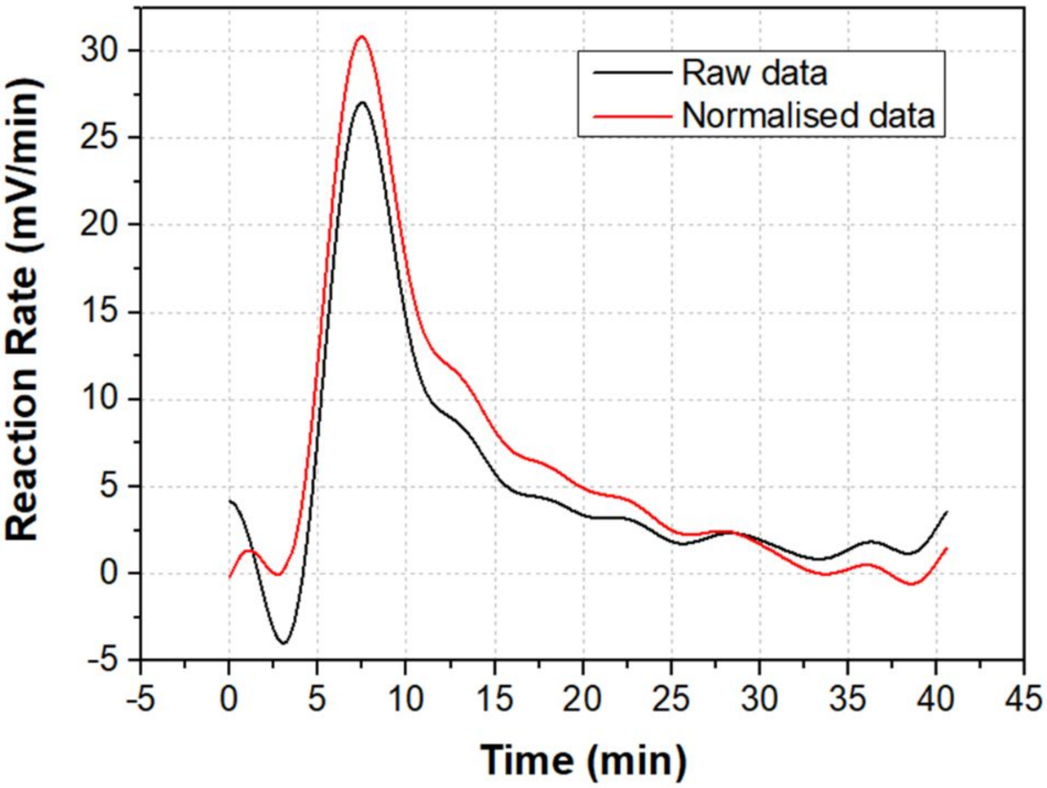

Figure S2: The impact of α_2M-thrombin can be subtracted mathematically for the TG curve because the concentration of α_2M is much higher (3–6 μM) than that of free thrombin (<500 nM) at any time and the overall consumption of α_2M during coagulation is limited (<0.5 μM). The velocity with which α_2M-thrombin is formed is therefore proportional to the concentration of thrombin. The rate constant of this reaction (k) is such as it is possible to obtain the stable end amidolytic activity. The k value was calculated and applied to the raw data (black curve) to get a normalized data (red curve) with the elimination of the impact of α2M-thrombin.

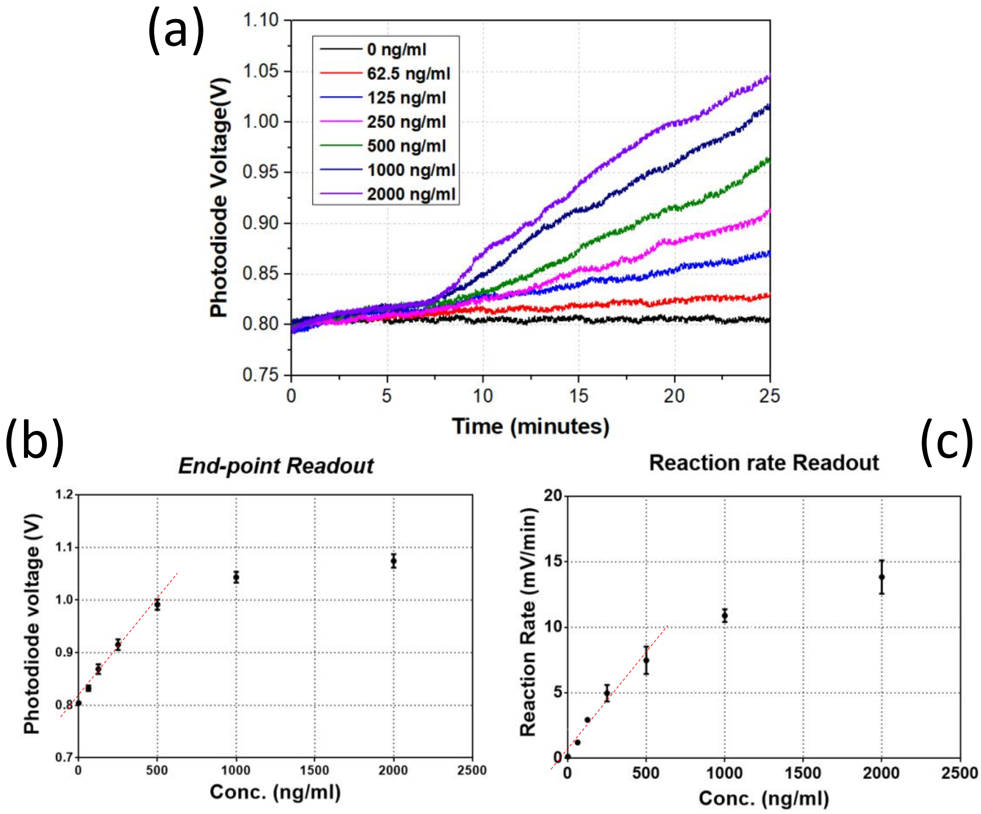

Figure S3: Results from the D-dimer assay with concentrations from 0 to 2000 ng/ml. (a) Reaction curves from the assay. (b) End-point readout. (c) Reaction rate readout at 17 minute after the reaction start. No obvious difference was found between the end-point and reaction rate readouts. Linear range was up to 500 ng/ml for both cases.

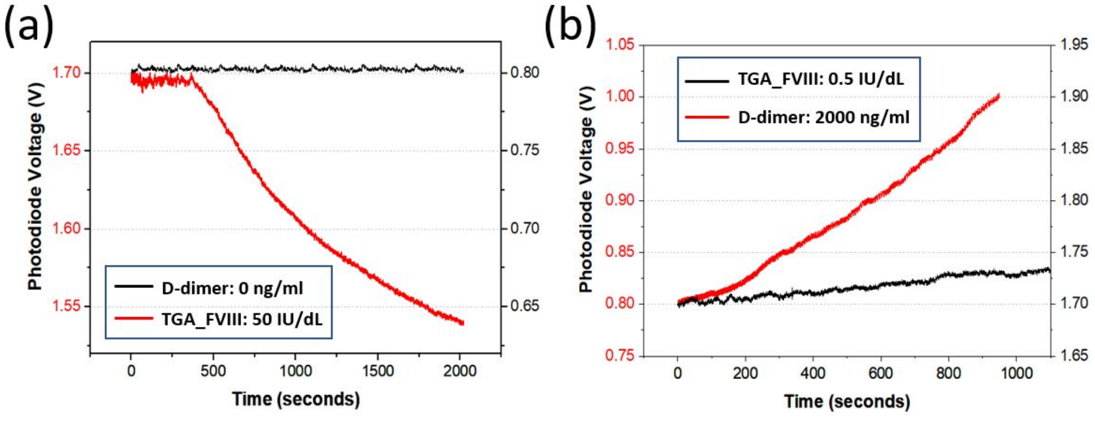

Figure S4: Cross-talk tests were undertaken to check the performance of the two reaction zones. (a) The plasma sample contains 50 IU/dL FVIII and no D-dimer. The expected signal decrease was recorded from the TGA reaction zone, and no signal change was detected from the D-dimer reaction zone. (b) The plasma sample contains no additional FVIII, but with 2000 ng/ml D-dimer. The expected signal increase was recorded from the D-dimer reaction zone, and expected signal change was detected from the TGA reaction zone. No obvious cross-talk issue was observed.

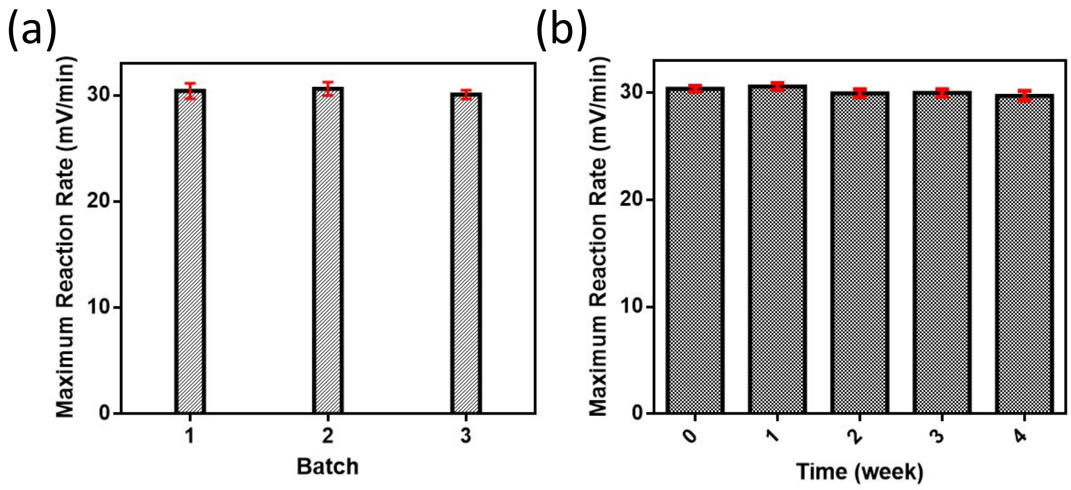

Figure S5: Reproductivity and stability of the device. (a) The reproductivity of the device was tested by comparing the maximum reaction rates among three different batches. (b) The stability of the device was tested by comparing the maximum reaction rates from different weeks.

Table S1: Comparison of the diagnostic methods for VTE

Diagnostic Tests	Target	Method	Accuracy	Stand alone	Sample	Cost	Operator
**CPTP**	VTE (DVT & PE)	Clinical predictio n rules	Low	No	n/a	Low	Very experience d expert
**D-dimer testing**	VTE (DVT & PE)	Blood test	Moderate	Yes	Human plasma	Mediu m	Expert
**CT & MRI venograph y**	DVT	Imaging	High	Yes (for DVT)	n/a	high	Expert
**CTPA**	PE	Imaging	Moderate to high	Yes (for PE)	n/a	high	Expert
**V/Q scan**	PE	Imaging/ scanning	Moderate	No	n/a	Mediu m	Expert
**D-dimer & TGA**	VTE (DVT & PE)	Blood test	High	Yes	Human plasma	Low	Non-expert

*CPTP: clinical pretest probability; CT: computerized tomography; MRI: magnetic resonance imaging; CTPA: computed tomography pulmonary angiography; V/Q: ventilation perfusion.
